# Divergent Regulation of CBF Regulon on Cold Tolerance and Plant Phenotype in Cassava Overexpressing Arabidopsis *CBF3* Gene

**DOI:** 10.3389/fpls.2016.01866

**Published:** 2016-12-06

**Authors:** Dong An, Qiuxiang Ma, Wei Yan, Wenzhi Zhou, Guanghua Liu, Peng Zhang

**Affiliations:** ^1^National Key Laboratory of Plant Molecular Genetics, CAS Center for Excellence in Molecular Plant Sciences, Institute of Plant Physiology and Ecology, Shanghai Institutes for Biological Sciences, Chinese Academy of ScienceShanghai, China; ^2^Institute of Tropical and Sub-tropical Cash Crops, Yunnan Academy of Agricultural SciencesBaoshan, China

**Keywords:** *Manihot esulenta* Crantz, CBF3, CBF regulon, cold and drought tolerance, phenotype change

## Abstract

Cassava is a tropical origin plant that is sensitive to chilling stress. In order to understand the CBF cold response pathway, a well-recognized regulatory mechanism in temperate plants, in cassava, overexpression of an Arabidopsis *CBF3* gene is studied. This gene renders cassava increasingly tolerant to cold and drought stresses but is associated with retarded plant growth, leaf curling, reduced storage root yield, and reduced anthocyanin accumulation in a transcript abundance-dependent manner. Physiological analysis revealed that the transgenic cassava increased proline accumulation, reduced malondialdehyde production, and electrolyte leakage under cold stress. These transgenic lines also showed high relative water content when faced with drought. The expression of partial CBF-targeted genes in response to cold displayed temporal and spatial variations in the wild-type and transgenic plants: highly inducible in leaves and less altered in apical buds. In addition, anthocyanin accumulation was inhibited by downregulating the expression of genes involved in its biosynthesis and by interplaying between the *CBF3* and the endogenous transcription factors. Thus, the heterologous *CBF3* modulates the expression of stress-related genes and carries out a series of physiological adjustments under stressful conditions, showing a varied regulation pattern of CBF regulon from that of cassava CBFs.

## Introduction

Cassava (*Manihot esculenta* Crantz) is widely cultivated for its starchy storage roots in tropics and subtropics where it provides a substantial source of staple food and animal feed (Cock, 1982; Hillocks et al., 2002). In China and other Southeast Asian countries, cassava is mainly used for starch, modified starch, and bioethanol production (Nguyen et al., 2007; Howeler, 2015). As a native to a warm habitat of tropics, cassava is adapted to the zone within a latitude 30° north and south of the equator and is thus, categorized as an extremely chilling-sensitive species (Alves, 2002; Huang et al., 2005). Therefore, low temperature is one of the major limiting factors for its geographical distribution and productivity, restricting its growth and survival below 18°C (An et al., 2012). With the cassava industrialization in China, expanding its cultivation has been promoted in subtropical regions, especially up to latitude 30°N (Jansson et al., 2009). Encountering the low temperature during late autumn, winter, and early spring, sometimes accompanied with extreme frozen climate is a major obstacle to high cassava yield and stem storage for propagation (An et al., 2012). Therefore, to acquire a prolonged growth period (i.e., early planting and late harvesting) in the high-latitude regions and a stable yield under stressful conditions, novel cassava cultivars with improved abiotic stress tolerance are in demand (Liu et al., 2011; Xu et al., 2014).

In order to cope with abiotic stresses that adversely affect the growth and yield, plants have evolved a set of adaptation mechanisms, including physiological and molecular processes (Bohnert et al., 1995; Ahuja et al., 2010). Expression of some stress-induced genes that might be involved in stress tolerance, transcription regulation, or signal transduction has been extensively studied in plants such as Arabidopsis and rice (Thomashow, 1999; Shinozaki et al., 2003; Chinnusamy et al., 2007; Nakashima et al., 2009). During transcriptional regulation, transcription factors (TFs) respond to stress stimuli via regulation of the downstream cascade of the target genes to protect the plant cells from injury. Among abiotic stress-regulated TFs, a small family of transcriptional activators known as dehydration-responsive element binding factors 1 (DREB1s)/C-repeat-binding factors (CBFs), including CBF1, CBF2, and CBF3 (also known as DREB1B, DREB1C, and DREB1A, respectively), were identified as the central regulators (Mizoi et al., 2012; Zhao et al., 2016). These factors bind to the low-temperature responsive DNA regulatory element termed C-repeat (CRT)/dehydration response element (DRE) of promoters in cold-inducible genes (Yamaguchi-Shinozaki and Shinozaki, 1994; Gilmour et al., 1998; Thomashow, 1999; Thomashow et al., 2001). In Arabidopsis, CBFs are essential for cold acclimation and freeze tolerance, which is controlled by regulating the expression of approximately 12% of the cold-responsive (*COR*) genes (Fowler and Thomashow, 2002) out of the recently confirmed 414 *COR* genes using CRISPR/Cas9 based *CBF* mutants (Zhao et al., 2016). Moreover, the constitutive expression of CBF genes induces the accumulation of mRNAs from genes that contain the CRT/DRE motif in their promoters, the CBF regulon, as well as from those which exhibit an increase in freezing and other abiotic stresses tolerance (Kasuga et al., 1999; Zhao et al., 2016). After being identified in Arabidopsis, many CBF homologs have been found in various plant species, including both dicots and monocots. These homologs have also demonstrated a key role in multiple stress response and tolerance by regulating the corresponding CBF-regulons (Jaglo et al., 2001; Hsieh et al., 2002a,b; Dubouzet et al., 2003; Ito et al., 2006; Chen et al., 2008). These results indicate that the upregulation of CBF expression is a feasible approach in improving multi-stress tolerance of agriculturally critical crops (Mickelbart et al., 2015).

C-repeat-binding factor cold response pathway is activated during cold acclimation of temperate plants, which not only causes metabolic changes including accumulation of soluble sugars and proline but also inhibits the plant cell growth through regulating different regulons (Gilmour et al., 2004; Park et al., 2015; Zhao et al., 2016). In addition to the improved stress tolerance, constitutive expression of CBFs in transgenic Arabidopsis, tomato, wheat, and barley exerts an adverse effect on plant phenotypes, such as growth retardation and yield reduction (Jaglo-Ottosen et al., 1998; Hsieh et al., 2002a,b; Morran et al., 2011). Thus, these phenotypic responses underlie the CBF interplay of the balance of cold stress regulation and plant growth (Zhao et al., 2016). The tropical plants, such as cassava, rice, maize, and tomato, are very sensitive to chilling stress and largely lack the capacity for cold acclimation (Zhu et al., 2007). The CBF orthologous gene *MeCBF1* of cassava has been proved to be induced by cold; nevertheless, compared with Arabidopsis CBFs, its retarded expression in response to cold and attenuated CBF-regulon might lead cassava to chilling sensitivity (An et al., Unpublished data). Therefore, understanding the CBF-mediated response to abiotic stresses in the non-cold-acclimating plants such as cassava may reveal the regulatory differences between temperate and tropical plants during evolution.

Herein, we report the improved cold and drought tolerance of transgenic cassava plants overexpressing Arabidopsis *CBF3* gene. Apparently, ectopic expression of *CBF3* modulates the expression of many stress-related genes, some of them belonging to CBF-regulons, subsequently resulting in increased accumulation of osmolytes and reduced cell damage under normal and stressful conditions. Intriguingly, growth retardation and downward leaf curling in the CBF3 overexpressing cassava indicate an altered CBF regulon that is regulated by the heterologous CBF gene.

## Materials and Methods

### Plasmid Construction and Cassava Transformation

The full-length CBF3 complementary DNA (cDNA) of Arabidopsis (*AtCBF3*) was firstly cloned into the pMD18-T vector (TaKaRa Biotechnology, Dalian, China). The released *AtCBF3* fragment was then cleaved by restriction endonucleases, *Kpn*I and *Pst*I, and inserted into the multiple cloning sites between CaMV 35S promoter and NOS terminator of a modified pCAMBIA1301 to generate the binary vector pC35S-AtCBF3 (Supplementary Figure 1). The construct was mobilized into *Agrobacterium tumefaciens* LBA4404, which was used for cassava transformation by friable embryogenic callus-mediated method (Zhang and Gruissem, 2004).

### Southern Blotting Analysis of Transgenic Cassava Plants

Genomic DNA was isolated from mature leaves of transgenic and wild-type (WT) cassava plants using the CTAB DNA extraction method. Twenty μg of total DNA was digested overnight with *Eco*RI. The fragmented product was separated on a 0.8% agarose gel and transferred onto a Hybond-N^+^ membrane (Amersham Biosciences, Piscataway, NJ, USA). The blot was hybridized with the DIG-labeled hygromycin phosphotransferase (HPT) gene probe and exposed to X-ray film for signal detection. The *HPT* probe was amplified by PCR using the following primers: 5′-TGAAAAAGCCTGAACTCACCG-3′ (forward) and 5′-TATTCTTTGCCCTCGGACG-3′ (reverse). The PCR program was as follows: 3 min at 94°C; 28 cycles of 30 s at 94°C, 30 s at 55°C, and 40 s at 72°C; followed by 10 min at 72°C; and finally stored at 4°C. DNA probe preparation, hybridization, and membrane washing were performed using DIG High Prime DNA Labeling and Detection Starter Kit II according to the manufacturer’s instructions (Roche, Grenzacherstrasse, Basel, Switzerland).

### Growth Conditions and Abiotic Stress Treatment of Cassava Plants

One-month-old *in vitro* seedlings of *AtCBF3* overexpressing lines (OE) and WT were planted into soil pots and grown in the greenhouse at 30°C/25°C with 16 h/8 h light/dark cycle and 30–40% relative humidity. For cold stress, 1-month-old plants grown in the greenhouse were exposed to 0°C for 24 h and then recovered for 1 month. For drought stress, stem-propagated pot-grown plants were cultivated in the greenhouse for 3 months under a fully watered regime (approximately 20% water content) and then the water was withdrawn for 21 days until the plants showed distinct symptoms of injury.

For field-grown cassava, about 6-month-old plants were subjected to seasonal changes. At the temperature below 15°C/8°C for day/night in November, the WT plants began to exhibit cold injury. During field growth, six different tissues of transgenic line OE-3 and WT, including apical buds, immature leaves, mature leaves, old leaves, petioles, and young stems, were harvested under three conditions of different day/night temperatures (September: 33°C/25°C as normal temperature; October: 25°C/16°C as cold treatment I, November: 15°C/8°C as cold treatment II), and frozen in liquid nitrogen for RNA extraction. Each treatment had more than three plants per line, and all treatments were conducted in three biological replicates.

### Measurements of Proline, Malondialdehyde (MDA), Electrolyte Leakage, and Relative Water Content (RWC)

One-month-old transgenic OE lines and WT plants were divided into two groups (normal growth and cold treatment), each group containing about 12 plants and each independent line consisting of at least three plants. After the plants had been exposed to 4°C for 12 and 24 h, approximately 0.5 g leaf tissue was harvested for each sample and extracted with various reagents according to different assays. Proline concentrations were measured by the sulfosalicylic acid-acid ninhydrin method (Bates et al., 1973). Malondialdehyde (MDA) equivalent was measured as described previously (Hodges et al., 1999). Electrolyte leakage was assessed following the method by Gilmour et al. (2000).

The RWC of cassava plants under normal growth conditions and drought stress was measured according to Gaxiola et al. (2001). The drought tolerance of these plants was evaluated by growing the plants for 3 months under a fully watered regime and then depleting water for about 3 weeks until these plants displayed obvious injury symptoms. The leaves from OE lines and WT were excised and their fresh weights recorded immediately. Their rehydrated weight was determined after floating the leaves in deionized water at 4°C overnight. Finally, they were dried in an oven at 70°C overnight and weighed. Each measurement was repeated three times. The RWC was calculated according to the formula: RWC = (fresh weight – dry weight)/(rehydrated weight – dry weight) × 100%.

### Semi-Quantitative and Real-Time RT-PCR Analysis

Total RNA was extracted from leaves of 1-month-old cassava plants grown under normal conditions or cold stress (4°C for 12 days) using TRIzol reagent according to the manufacturer’s protocol (Invitrogen). The RNA quality was determined on an agarose gel by ethidium bromide staining. Reverse transcription was performed using the ReverTra Ace qPCR RT Kit according to the manufacturer’s protocol (TOYOBO, Osaka, Japan). The cDNA was directly used as templates for semi-quantitative RT-PCR or diluted fivefold for real-time RT-PCR amplification on a Bio-Rad CFX96 thermocycler (CFX96 thermocycler (Hercules, CA, USA) with gene-specific primers (Supplementary Table 1). The quantitative variation between different samples was evaluated by the 2^-ΔΔCt^ method, and cassava β-actin was used as an internal control. All of the samples were measured in triplicate, and the experiments were performed on three biological replicates.

### Statistical Analysis

All data were determined from at least three biological replicates and are presented as mean ± SD. Asterisks indicate significant differences compared with the WT at ^∗^*P* < 0.05 or ^∗∗^*P* < 0.01 by Student’s *t*-test.

## Results

### Overexpression of Arabidopsis CBF3 Gene in Cassava

**FIGURE 1:**
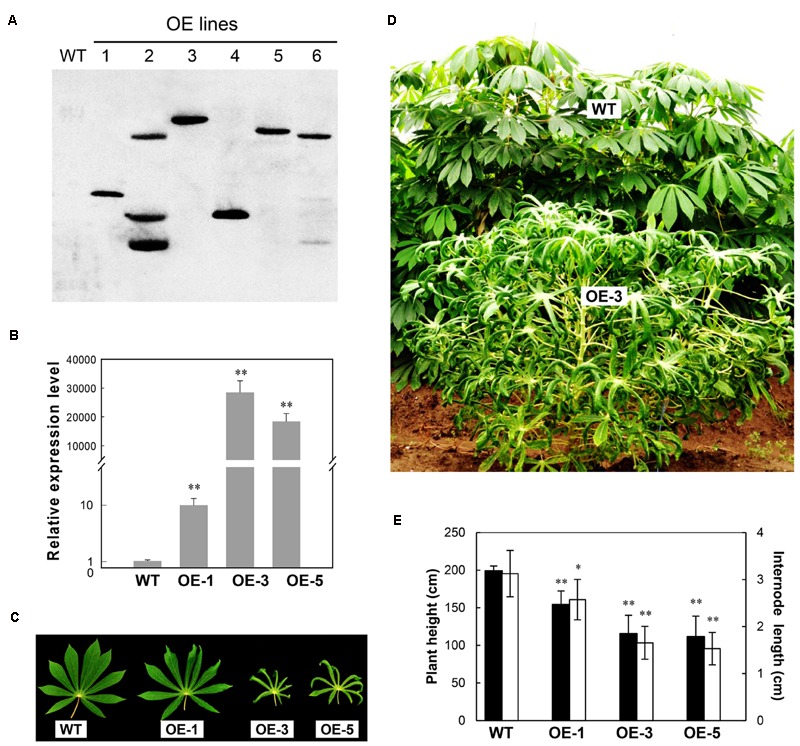
**Molecular and phenotypic characterization of Arabidopsis *CBF3* overexpressing cassava. (A)** Transgene integration pattern in wild-type (WT) and transgenic (OE) lines by Southern analysis. **(B)** Quantitative analysis of the DREB1A/CBF3 expression in WT and three single-copied transgenic lines (OE-1, OE-3, and OE-5) by real-time RT-PCR. **(C)** Leaves collected from field-grown plants. **(D)** Plant status in the field by comparison of WT and OE-3 plants. **(E)** Plant height and internode length between WT and OE plants. Data are presented as mean ± SD of three independent assays; asterisks indicate significant differences compared with the WT, ^∗^*P* < 0.05 or ^∗∗^*P* < 0.01 by Student’s *t*-test.

Six *AtCBF3* overexpressing (OE) transgenic cassava plant lines regenerated from transformed friable embryogenic calli were multiplied by shoot cultures *in vitro*. Their transgene integration patterns were verified by the DIG-labeled *HPT* probe using Southern blotting analysis. Four out of six OE lines were identified as single copy insertion into the genome by *Eco*RI digestion (**Figure 1A**). Their AtCBF3 transcript level initially analyzed by semi-quantitative RT-PCR confirmed the high expression of the genes in three lines, OE-1, OE-3, and OE-5 (Supplementary Figure 2). OE-3 and OE-5 lines showed a remarkable overexpression of the CBF3 gene, approximately several 1000-fold higher than that of the OE-1 line, by real-time RT-PCR analysis (**Figure 1B**). Therefore, these three lines together with WT were used for subsequent studies.

**FIGURE 2:**
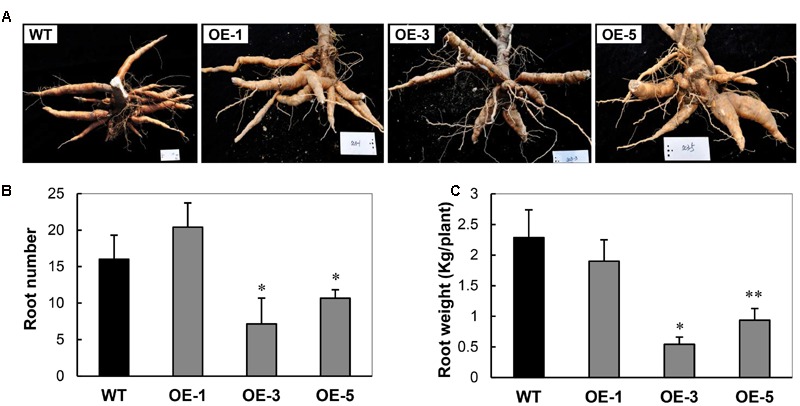
**Characteristics of storage roots harvested from the field-grown wild-type (WT) and transgenic (OE) lines. (A)** Storage root phenotypes. **(B)** Storage root number per plant. **(C)** Storage root weight per plant. Data are presented as mean ± SD of three independent assays; asterisks indicate significant differences compared with the WT, ^∗^*P* < 0.05 or ^∗∗^*P* < 0.01 by Student’s *t*-test.

Similar to the most transgenic plant lines overexpressing CBF subfamily members that showed undesired side-effects such as severe growth retardation, the OE cassava lines also displayed downward leaf curling, especially in OE-3 and OE-5 (**Figure 1C**), and severe retardation under normal growth conditions in the field (**Figure 1D**). Plant height and internode length of the OE lines were significantly shorter than those of WT (**Figure 1E**), about 50%. Upon harvest, their phenotypes of storage roots were photographed (**Figure 2A**). Noticeably, the storage root number and yield of OE-3 and OE-5 were reduced to 30–50% of WT (**Figures 2B,C**). The OE-1 plants showed less change in these traits. These phenotypic changes were obviously dependent on the transcript abundance of the *AtCBF3* gene.

### Expression of Arabidopsis *CBF3* Enhances Cassava Tolerance to Cold Stress

**FIGURE 3:**
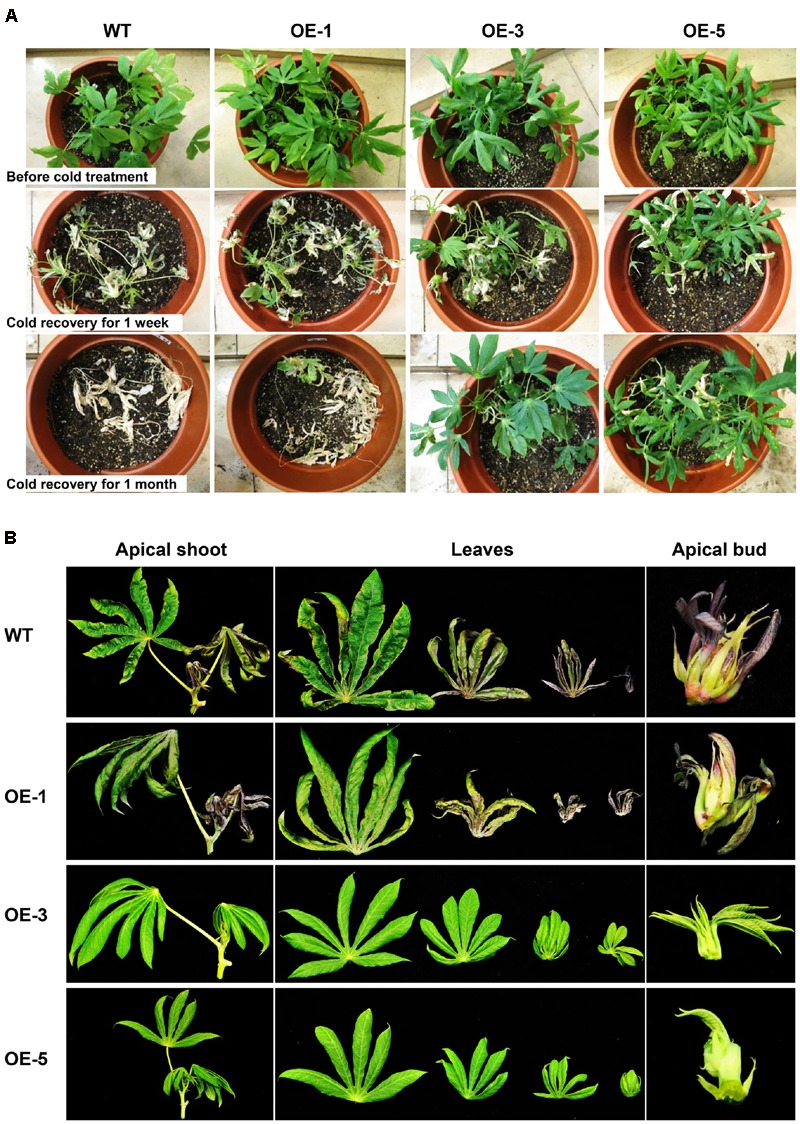
**Performance of Arabidopsis *CBF3* overexpressing cassava in response to low temperature. (A)** Pot-grown plants in the greenhouse before and after cold treatment (0°C, 24 h) for 1 week and 1 month recovery under normal growth condition. **(B)** Apical shoots, leaves, and apical buds harvested from WT and OE transgenic plants in the field under chilling condition in early November.

To investigate whether overexpression of *AtCBF3* confers abiotic stress tolerance in cassava, 1-month-old OE and WT seedling plants grown in the greenhouse were exposed to cold stress (0°C) for 24 h, followed by recovery of the growth under standard conditions (25°C). The OE transgenic plants recuperated better than the WT (**Figure 3A**). In particular, after 1 month, the OE-3 and OE-5 lines continued with a vigorous growth, including sprouting new leaves; while OE-1 plants only survived. Contrastingly, WT plants could not be recovered and finally perished.

In the field, under the seasonal transition from autumn to early winter, the phenotypic exhibition of these plants was also different. When the temperature decreased to 15°C/8°C (day/night) at the beginning of November, WT plants displayed obvious cold injury symptoms. Their apical shoots including apical buds and immature leaves were dehydrated and yellowish with a loss of vitality. Moreover, the OE-3 and OE-5 lines showed normal foliage status as before (**Figure 3B**). The shoots of OE-1 plants were also affected by the low temperature but less damaged as compared to those of WT. The observation indicates that the high overexpression of *AtCBF3* in cassava plants increases their tolerance to low-temperature stress in a dose-dependent manner.

### *AtCBF3* Overexpression Protects Cassava against Cold-Induced Cell Damage

**FIGURE 4:**
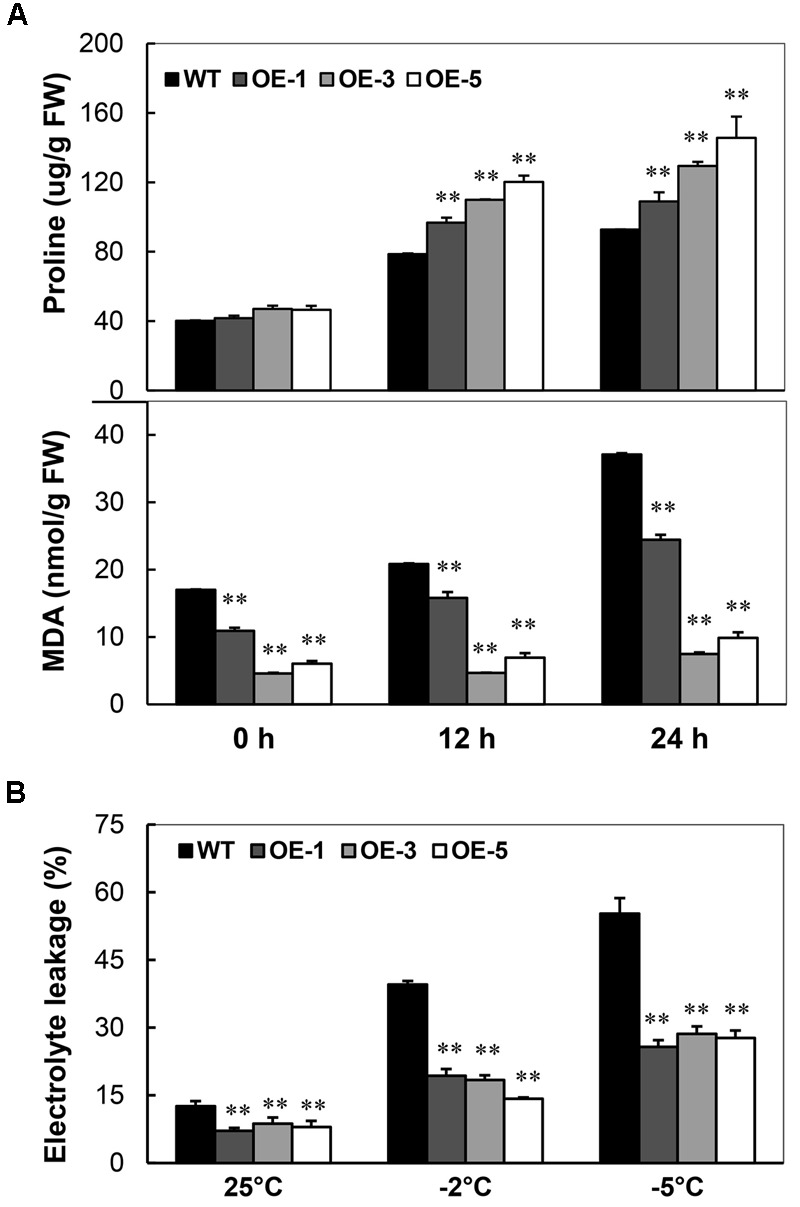
**Changes of proline content, MDA level, and electrolyte leakage in Arabidopsis *CBF3* overexpressing cassava. (A)** Proline concentration and MDA level in the WT and transgenic OE plants under cold treatment (-2°C) for 0, 12, and 24 h. FW, fresh weight. **(B)** Electrolyte leakage of WT and OE plants before and after cold treatment (-2 and -5°C) for 12 h. Data are presented as mean ± SD of three independent assays; asterisks indicate significant differences compared with the WT at ^∗∗^*P* < 0.01 by Student’s *t*-test.

Under the normal condition, the content of proline in seedlings of OE lines was similar to that of WT. A significant increase of proline content was detected in the OE lines than that of WT under -2°C stress (**Figure 4A**, upper panel) with a maximum of 52.9% increase at 12 h and 57% at 24 h. Strikingly, the MDA contents were always lower in OE lines than that in WT. Upon treatment, the minimum level of MDA in OE lines was about 26.9% that of WT; after 24 h treatment, the minimum level of MDA in OE lines only reached about 20.2% that of WT (**Figure 4A**, lower panel). Similarly, the electrolyte leakage in the leaf disks was also less in OE lines than WT: about half of WT after -5°C treatment (**Figure 4B**). These results indicate that the overexpression of *AtCBF3* protects the cassava with less free radical production and from cellular membrane injury caused by cold stress.

### AtCBF3 Activates Many Stress-Related Genes in Transgenic Cassava

In order to speculate whether the AtCBF3 improves the abiotic stress tolerance of transgenic cassava through CBF cold-responsive pathway and the endogenous CBF-regulon response to stress, the transcript profiles of potential CBF-targeted genes (**Table 1**) were analyzed. These profiles were assessed using the apical bud, young leaf, mature leaf, old leaf, petiole, and young stem of field-grown WT and OE-3 plants under three different day/night temperatures (September 33°C/25°C as normal temperature, T1; October 25°C/16°C as cold treatment I, T2; November 15°C/8°C as cold treatment II, T3). The homologous genes in the cassava genome sequence from Phytozome^1^ were discovered by protein sequence alignment with Arabidopsis stress-related genes. Several homologous genes, including *MeRAP2.1, MeERD7, MeCOR47, MeKIN2, MeGolS3, MeLTI30, MeLEA14, MeOEP16, MeCOR78*, and *MeATDI21*, were identified and their promoter regions ca. 2000 bp length were cloned. Six out of 10 genes belonged to the CBF-target genes as they harbored the CBF binding element DRE/CRT (G/ACCGAC, Yamaguchi-Shinozaki and Shinozaki, 1994; Stockinger et al., 1997). The other three genes, whose promoters did not contain this element, might be regulated by CBF3 via other *cis*-elements or other regulators mediated by CBF3. However, the promoter of *MeCOR78*, a *COR* gene, was not found (**Table 1**).

**Table 1:** CBF3-targeted orthologous genes in cassava.

Gene name	Accession number	DRE/CRT (G/ACCGAC)	Annotation
*MeATDI21*	JQ807808	-790 to -785: ACCGAC	Stress-inducible protein
*MeCOR15b*	JQ807804	-1558 to -1553: GCCGAC	Cold regulated gene
*MeCOR47*	JQ807801	-548 to -543: GCCGAC	Dehydrin
*MeCOR78*	JQ807806	Not found	Cold regulated gene
*MeERD7*	JQ807799	No DRE/CRT	Early drought-inducible gene
*MeFAD7*	JQ807800	No DRE/CRT	Fatty acid desaturase 7
*MeGolS3*	JQ807802	-167 to -163: CCGAC	Galactinol synthase
*MeKIN2*	JQ807805	No DRE/CRT	Cold responsive 6.6
*MeLEA14*	JQ807798	-1955 to -1951: CCGAC	Late-embryogenesis abundant protein
*MeLTI30*	JQ807797	No DRE/CRT	Dehydrin
*MeOEP16*	JQ807803	-432 to -427: GCCGAC	16-KDa plastid outer membrane protein
*MeRAP2.1*	JQ807807	-35 to -31: CCGAC	Encodes a member of the DREB subfamily; A-5 of ERF/AP2 transcription factor family

**FIGURE 5:**
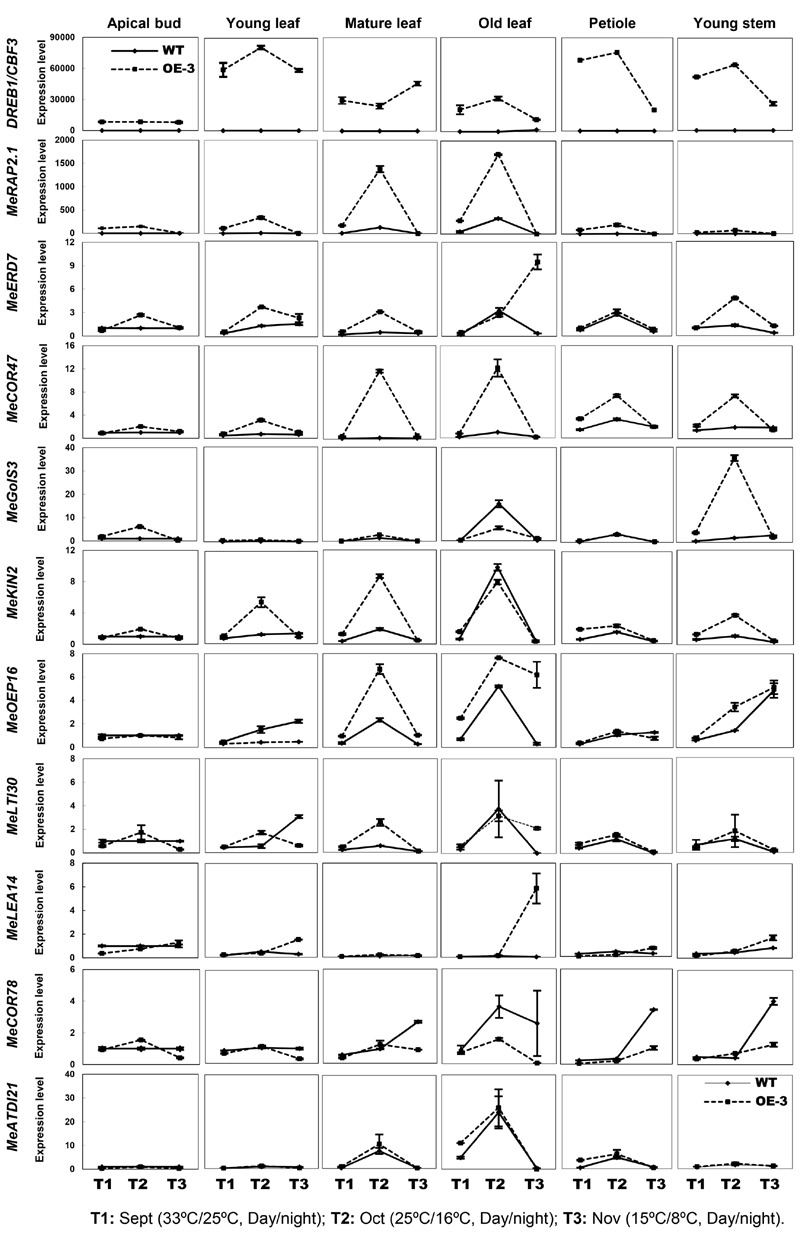
**Expression patterns of Arabidopsis *CBF3* and alleged members of CBF-regulon in cassava under the seasonal temperature changes.** Six different tissues of WT and the OE-3 transgenic plants were used, including apical bud, young leaf, mature leaf, old leaf, petiole, and young stem. Description of cassava genes is listed in **Table 1**.

Compared to WT, all tissues of the OE-3 line detected an increased expression of CBF3, the highest in young leaf, petiole, and young stem, whereas the apical bud showed the lowest expression of the gene (**Figure 5**). Interestingly, all the stress-related genes exhibited little expression changes in the apical bud of both WT and OE-3 plants indicating an unknown upstream factor that might be inhibiting the expression induction of these stress-response genes in cassava meristematic tissues. In other tissues, most of the genes showed upregulated expression by the cold treatment I (T2) and maintained similar levels as WT by cold treatment II (T3) in the OE-3 line. Nevertheless, specific response of these genes to cold treatment varies among different tissues. For example, in the young and mature leaf of OE-3 line, the expression of *MeRAP2.1, MeERD7, MeCOR47*, and *MeKIN2* was obviously induced by the cold treatment I (T2); however, the expression of these genes was less affected by the treatment in WT. The expression of *MeGolS3* only changed in the young stem by T2. Several genes whose promoters lacked the DRE/CRT element, especially in *MeERD7, MeLTI30*, and *MeCOR78* showed less difference in expression between the WT and OE-3 plants under the normal or cold treatments. The expression of other genes such as *MeLEA14* and *MeATDI21*, containing the DRE/CRT element in their promoters was not affected by the overexpression of *AtCBF3* (**Figure 5**), indicating the variations between Arabidopsis and cassava CBF regulons. Taken together, cassava CBF regulon differed from that of Arabidopsis and its regulation showed temporal and spatial patterns in response to heterologous CBF and cold treatment.

**FIGURE 6:**
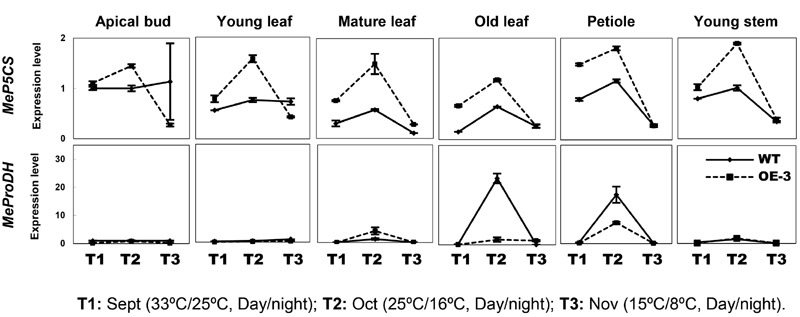
**Expression patterns of *MeP5CS* and *MeProDH* in cassava under the seasonal temperature changes.** Six different tissues of WT and the OE-3 transgenic plants were used, including apical bud, young leaf, mature leaf, old leaf, petiole, and young stem.

Since cold treatment promoted excessive proline accumulation in OE lines (**Figure 4A**), the expression profiles of *P5CS* (Δ1-pyrroline-5-carboxylate synthase) and *ProDH* (proline dehydrogenase), two key genes controlling the balance of biosynthetic and catabolic pathways (Sharma et al., 2011), were also checked in these different tissues. Upregulation of *MeP5CS* and downregulation of *MeProDH* were detected in the transgenic OE-3 line compared with those of WT under normal and cold treatment conditions (**Figure 6**). This suggested that ectopic overexpression of *AtCBF3* might favor the modulation of stress-related gene expression in response to abiotic stress in transgenic cassava.

### Constitutive Expression of *AtCBF3* Enhances Cassava Tolerance to Drought

**FIGURE 7:**
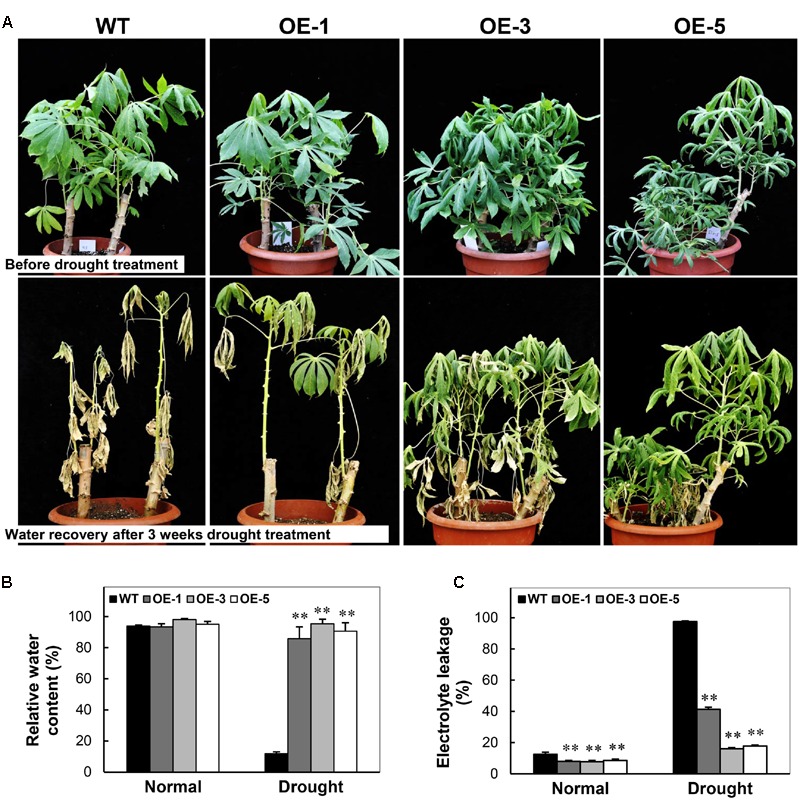
**Performance of Arabidopsis *CBF3* overexpressing cassava in response to drought. (A)** 3-month-old pot-grown plants in the greenhouse before and after drought treatment, i.e., withheld water for 3 weeks. **(B,C)** Relative water content **(B)** and electrolyte leakage **(C)** of the WT and OE lines under normal and drought stress. Data are presented as mean ± SD of three independent assays; asterisks indicate significant differences compared with the WT at ^∗∗^*P* < 0.01 by Student’s *t*-test.

To examine the drought stress tolerance of OE transgenic cassava, the 3-month-old plants of OE lines and WT grown in pots were subjected to drought treatment by water depletion. Before drought stress, all the plants exhibited active growth status (**Figure 7A**). After water depletion for 3 weeks, WT plants showed distinct injury phenotype including severe wilting and dehydrated leaves. Contrastingly, the OE plants consisted of more green leaves and relatively less dehydration phenotype (**Figure 7A**), especially in OE-3 and OE-5 plants. Their RWC was dramatically higher than WT, about ninefold after the drought treatment, although no difference was recorded between the OE and WT plants under normal growth conditions (**Figure 7B**). Furthermore, the electrolyte leakage of leaves in OE plants was reduced significantly than that in WT under normal growth conditions. Under drought stress, WT showed approximately 90% electrolyte leakage, while that of OE lines was only 15–40% (**Figure 7C**). The phenotype indicates that the integrity of the membrane was dramatically injured in WT but preserved in OE lines. These results demonstrate that the overexpression of *CBF3* resulted in improved drought stress tolerance in transgenic cassava plants.

### AtCBF3 Negatively Regulates Anthocyanin Biosynthesis in Transgenic Cassava

**FIGURE 8:**
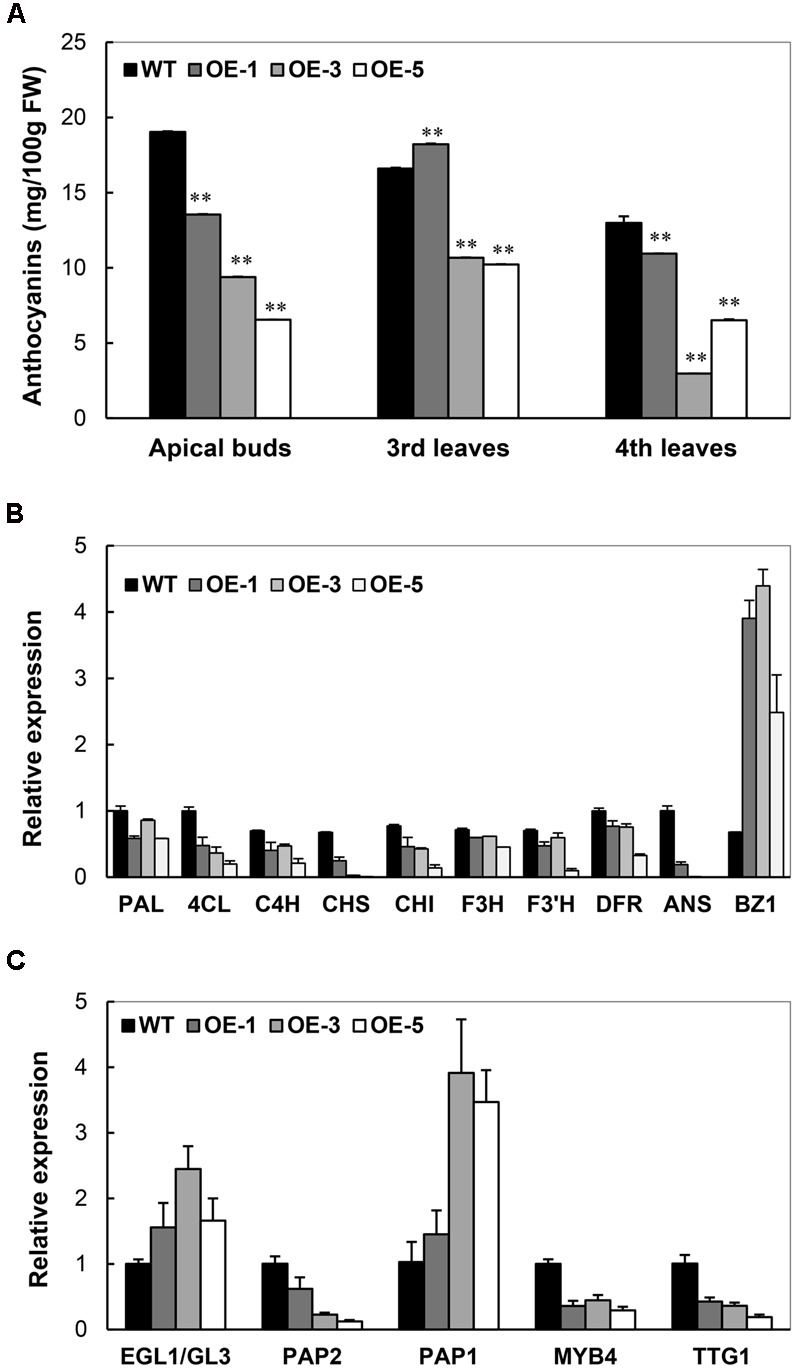
**Characterization of anthocyanin biosynthesis in Arabidopsis *CBF3* overexpressing cassava. (A)** Changes of anthocyanin content in the apical buds, the third and fourth leaves from the top of WT and OE plants. **(B)** Expressional changes of genes related to anthocyanin biosynthesis. PAL, phenylalanine ammonia-lyase; 4CL, 4-coumarate-CoA ligase; C4H, *trans*-cinnamate-4-monooxygenase; CHS, naringenin-chalcone synthase; CHI, chalcone isomerase; F3H, naringenin 3-dioxygenase; F3′H, flavonoid 3′-hydroxylase; F3′5′H, flavonoid 3′,5′-hydroxylase; DFR, dihydroflavonol4-reductase; ANS, anthocyanidin synthase; BZ1, anthocyanidin 3-*O*-glucosyltransferase. **(C)** Expressional changes of cassava transcription factors (EGL1/GL3, PAP2, PAP1, MYB4, and TTG1) that regulate anthocyanin biosynthesis.

As illustrated in **Figure 3B**, the absence of anthocyanin accumulation was noticed in the OE plants, especially OE-3 and OE-5. In cassava TMS60444, apical buds, young leaves, and the base region of petioles show the purple color of anthocyanins. A quantitative analysis of anthocyanins from apical buds and young leaves demonstrated a significant decrease of anthocyanins in the OE lines (**Figure 8A**). In their top leaves, the expression level of several anthocyanin biosynthetic genes in cassava was substantially decreased, including 4CL, CHS, CHI, and ANS. In addition, only the BZ1 gene showed an upregulated expression, possibly due to the feedback regulation (**Figure 8B**). In response to the downregulated expression of these genes, the expression of several TFs, such as MYB, MYC, WD-40-like that have been reported to regulate anthocyanin biosynthesis (Petroni and Tonelli, 2011), was also analyzed. Among these TFs, MePAP2 and MeTTG1 showed about 50% reduction in their expression (**Figure 8C**), although their promoter regions did not harbor the DRE/CRT element (data not shown).

## Discussion

The CBF regulatory pathway is well established as the core component of cold response controlling the expression of several *COR* genes in Arabidopsis and temperate crops (Chinnusamy et al., 2007; Zhao et al., 2016). In the present study, we generated transgenic cassava plants overexpressing Arabidopsis *CBF3* gene and found that such plants showed improved stress tolerance to cold and drought (**Figures 3** and **7**). Consequently, the *AtCBF3*-overexpressing cassava, under normal and stress conditions, showed many physiological alterations such as improved accumulation of proline, reduced membrane injury, and increased RWC, compared to those of WT. Similar results have been reported in transgenic plants overexpressing Arabidopsis *CBF3* (Nakashima et al., 2009). These results indicated that Arabidopsis CBF3 is functional and plays a similar role in enhancing tolerance to abiotic stress in the tropical species.

The expression of the corresponding CBF-regulon is essential for plants responding to various stresses and for the development of stress tolerance (Chinnusamy et al., 2004; Nakashima et al., 2009; Zhao et al., 2016). In Arabidopsis, CBF-regulon encompasses hundreds of genes that are involved in multiple functions, for example, *RAP2.1, COR47, OEP16, Gols3*, and *LEA14*. These members of CBF-regulon are stress-responsive and play vital roles in enhancing abiotic stress in Arabidopsis (Novillo et al., 2007; Park et al., 2015). CBF regulons are also species-specific and show variation between the temperate and tropical plants (Zhang et al., 2004; Carvallo et al., 2011). Although CBF-regulon has not yet been fully studied in cassava, among the 11 putative CBF-targeted genes identified in cassava genome (**Table 1**), most of them were highly inducible by the cold treatment in certain tissues, for example, leaves. Interestingly, the inducibility is significantly enhanced in the *AtCBF3*-overexpressing cassava for some genes, indicating that the CBF cold-responsive pathway is repressed up to some extent under cold stress and could be activated by AtCBF3. These CBF regulon members showed a strong temporal and spatial expression in cassava, out of which some were totally unresponsive to cold treatment, implying the difference between cassava and Arabidopsis CBF-regulons.

Our result of cold treatment in field-grown plants also revealed the response collapse of CBF regulons under the extended cold treatment (T3 treatment). Such treatment caused the cell damage in various tissues of cassava plants, leading to the reduced expression of CBF-targeted genes. Recently, we found that the retarded MeCBF1 expression in response to cold and attenuated CBF-regulon might be responsible for the chilling sensitivity of cassava (An et al., Unpublished data). It is also in agreement with our previous findings that the cassava apical bud is more sensitive to cold stress due to the CBF regulon (An et al., 2012). With the advances in the cassava genome sequencing (Wang et al., 2014; Bredeson et al., 2016), the screening of the key TFs that dominate the cold-response pathway in cassava is plausible using multiple approaches.

There are several obvious phenotypic changes in the *AtCBF3*-overexpressing cassava: one is the retarded plant growth with leaf curling, another is reduced anthocyanin accumulation, and the third is reduced storage biomass. The phenotypic changes may be caused by the altered expression of downstream *COR* genes or TFs. Several studies have reported that the overexpression of Arabidopsis CBFs might lead to retarded plant growth with leaf curling in Arabidopsis, tomato, tobacco, rice, and wheat under unstressed conditions. This phenomenon may be mediated through putative interaction with other TFs that have a growth suppressing role in order to disturb the regulation of leaf morphogenesis (Jaglo-Ottosen et al., 1998; Gilmour et al., 2000; Hsieh et al., 2002b; Kasuga et al., 2004; Pellegrineschi et al., 2004). When cassava overexpresses the native MeCBF1 gene, less effect on the leaf and plant growth is observed (An et al., Unpublished data), indicating that cassava evolved with the different regulatory mechanism of CBFs. The overexpression of *AtCBF3* in cassava also influences the expression of other TFs that regulate anthocyanin biosynthesis (**Figure 8C**), leading to reduced anthocyanin accumulation. On the other hand, the ectopic expression of a peach CBF in apple can increase anthocyanin accumulation (Wisniewski et al., 2011). All these characteristics suggest that the regulation and function of CBF may differ from others among plant species.

In summary, the ectopic expression of Arabidopsis CBF3 in cassava improves the plant performance against cold and drought. This reflects the conserved function of CBF in plants. The altered expression profiles in response to cold showed that the CBF-regulons in WT and transgenic cassava were different. Dramatic changes in the plant phenotype and downregulation of anthocyanin biosynthesis indicate the specific interplay of CBF3 with other native TFs or pathways, proving the functional divergence of CBF gene in evolution.

## Genbank Accession Numbers of Cassava Genes

JQ807797 (*MeLTI30*), JQ807798 (*MeLEA14*), JQ807799 (*MeERD7*), JQ807800 (*MeFAD7*), JQ807801 (*MeCOR47*), JQ807802 (*MeGolS3*), JQ807803 (*MeOEP16*), JQ807805 (*MeKIN2*), JQ807806 (*MeCOR78*), JQ807807 (*MeRAP2.1*), JQ807808 (*MeATDI21*), JQ807809 (*MeP5CS*), JQ807810 (*MeProDH*).

## Author Contributions

DA performed most of the experiments and drafted the manuscript. QM produced transgenic cassava. WY, GL, and WZ conducted part of field experiments and analysis. PZ coordinated and designed the study and revised the article.

## Conflict of Interest Statement

The authors declare that the research was conducted in the absence of any commercial or financial relationships that could be construed as a potential conflict of interest.

## Footnotes

1 https://phytozome.jgi.doe.gov/Mesculenta
