# Supplementary material for: Divergent Regulation of CBF Regulon on Cold Tolerance and Plant Phenotype in Cassava Overexpressing Arabidopsis CBF3 Gene
Source: Front Plant Sci. 2016 Dec 6;7:1866. doi: 10.3389/fpls.2016.01866 (PMC5138201; doi:10.3389/fpls.2016.01866)
Supplement: Supplementary file 1 [file Presentation_1.PDF]

Supplementary Material

Divergent regulation of CBF regulon on cold tolerance and plant phenotype in cassava overexpressing Arabidopsis *CBF3* gene

Dong An, Qiuxiang Ma, Wei Yan, Wenzhi Zhou, Guanghua Liu, Peng Zhang*

*** Correspondence:** Corresponding Author: zhangpeng@sibs.ac.cn

Supplementary Table 1 The primers used in our study were listed.

Gene	Primer name	Sequence (5'→3')
MeATDI21	MeATDI21-Forward	CTAAGCTTCTCGTCGATGGTC
	MeATDI21-Reverse	CTGTGATGGGATCTGGAGC
AtCBF3	CBF3-full-length-Forward	GAGGATCCCTCTTCTGATCAATGAAC
	CBF3-full-length-Reverse	GAGAGCTCCTGAGTTTTAATAACTCC
	CBF3-partial-Forward	CGAGTCTTCGGTTTCCTCAG
	CBF3-partial-Reverse	TCTCCGACGAACTCCTCTGT
MeCOR47	MeCOR47-Forward	GAAGCCTGAAGAGGTAGCTACGC
	MeCOR47-Reverse	TGAACATGCTCAGTTGGTGGTGGC
MeCOR78	MeCOR78-Forward	TGGTGGTGAAAGGCAAAGCAG
	MeCOR78-Reverse	CGTTATTACAGCAGGAGGCTC
MeERD7	MeERD7-Forward	TAGCGTCGAGCTCGCTTGTG
	MeERD7-Reverse	CGAGCGAGAACGGCAACGAT
MeFAD7	MeFAD7-Forward	ACAGCCTGTTGGACAGCAATGGC
	MeFAD7-Reverse	GTACCAGGGAAGTTTGTCCTCATG
MeGOLS3	MeGOLS3-Forward	GCTTACGTGACTTTCTTGGCTG
	MeGOLS3-Reverse	CCTTTGGCCAACCCAACAAC
MeKIN2	MeKIN2-Forward	CGTGCCTCACTCATCTAGTAG
	MeKIN2-Reverse	CTTGCGACCTGCGTTGGAGT
MeLEA14	MeLEA14-Forward	GAGGGACATTGGCACAGACT
	MeLEA14-Reverse	GAGATCAGAAAGGCCAGGTG
MeLTI30	MeLTI30-Forward	CAACTGACGGATGAACACGG
	MeLTI30-Reverse	CCTTGGATGTGTGATCTGTTCC
MeOEP16	MeOEP16-Forward	TGGAGAGGGTTCGTGGCAC
	MeOEP16-Reverse	GTTGCAGCAGTCGCAATGGC
MeP5CS	MeP5CS-Forward	CGGACAGGTAGTGGATGGC
	MeP5CS-Reverse	GTGAATGCCAACGTCTCCAC
MeProDH	MeProDH-Forward	CCATCACCACACCTCCGTC
	MeProDH-Reverse	CACCACAGGGTTCATGGCAG
MeRAP2.1	MeRAP2.1-Forward	TCAGCTTCCGCCACCACAAC
	MeRAP2.1-Reverse	AACAGCGGTGTCGTAGGCAC

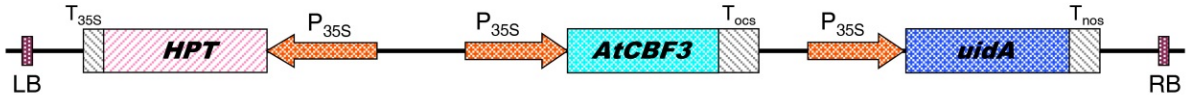

Supplementary Figure 1. Schematic representation of the T-DNA region of binary vector pC35S-AtCBF3. P_{35S}, CaMV 35S promoter; T_{35S}, CaMV 35S terminator; T_{ocs}, octopine synthase terminator; LB, left border; RB, right border; *HPT*, hygromycin phosphortransferase gene; *AtCBF3*, Arabidopsis CBF3 gene; *uidA*, β-glucuronidase gene..

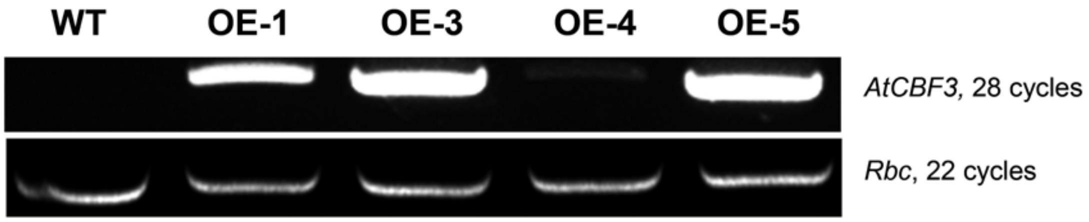

Supplemental Figure 2 Semi-quantitative RT-PCR analysis of Arabidopsis *CBF3* gene in the WT and OE transgenic plants. Rubisco large subunit (*Rbc*) gene was used as a reference.
